# Ethics of COVID-19-related school closures

**DOI:** 10.17269/s41997-020-00396-1

**Published:** 2020-08-07

**Authors:** Michael Silverman, Robert Sibbald, Saverio Stranges

**Affiliations:** 1 Division of Infectious Diseases, Western University, London, Ontario Canada; 2 Department of Epidemiology and Biostatistics, Western University, London, Ontario Canada; 3 Division of Medical Bioethics, Department of Family Practice, London Health Sciences Centre, London, Ontario Canada; 4 Department of Family Medicine, Western University, London, Ontario Canada; 5 Department of Population Health, Luxembourg Institute of Health, Strassen, Luxembourg

**Keywords:** COVID-19, School closure, Ethics, Child, COVID-19, fermeture d’école, éthique, enfant

## Abstract

COVID-19 mitigation strategies have led to widespread school closures around the world. Initially, these were undertaken based on data from influenza outbreaks in which children were highly susceptible and important in community-wide transmission. An argument was made that school closures were necessary to prevent harm to vulnerable adults, especially the elderly. Although data are still accumulating, the recently described complication, pediatric multisystem inflammatory syndrome, is extremely rare and children remain remarkably unaffected by COVID-19. We also do not have evidence that children are epidemiologically important in community-wide viral spread. Previous studies have shown long-term educational, social, and medical harms from school exclusion, with very young children and those from marginalized groups such as immigrants and racialized minorities most affected. The policy and ethical implications of ongoing mandatory school closures, in order to protect others, need urgent reassessment in light of the very limited data of public health benefit.

## Résumé

Les stratégies d’atténuation de la COVID-19 ont mené à des vagues de fermetures d’écoles dans le monde entier. Au départ, ces fermetures ont été décidées d’après les données sur les éclosions grippales, durant lesquelles les enfants sont très susceptibles et jouent un rôle important dans la transmission communautaire. On a fait valoir que les fermetures d’écoles étaient nécessaires pour protéger les adultes vulnérables, surtout les personnes âgées. Les données s’accumulent encore, mais le syndrome inflammatoire multisystémique chez les enfants, une complication décrite récemment, est extrêmement rare, et les enfants demeurent remarquablement peu touchés par la COVID-19. Nous n’avons pas non plus de preuves de leur importance épidémiologique dans la propagation communautaire du virus. Par contre, selon des études antérieures, l’exclusion scolaire cause des préjudices à long terme sur le plan éducatif, social et médical, et les très jeunes enfants et ceux des groupes marginalisés, comme les immigrants et les minorités racisées, en sont les plus touchés. Les conséquences stratégiques et éthiques de la prolongation des fermetures d’écoles obligatoires pour protéger les autres doivent être réévaluées d’urgence, vu les données très limitées sur les avantages que cela représente pour la santé publique.

Approximately 1.2 billion school children have had their education put on hold due to COVID-related school closures (UNESCO 2020). In fact, between 27/03/2020 and 22/04/2020, more than 90% of the total enrolled world-wide learners were subject to country-wide school closures and confined at home (UNESCO 2020).

Initial widespread closing of schools was undertaken under an understandable abundance of public health caution, in light of the severity of the epidemic and using the experience of influenza where young children are specifically vulnerable and are known to spread the virus effectively. In contrast to influenza, studies have consistently demonstrated that a disproportionate minority of COVID-19 cases have occurred in children, with some showing that they make up as few as 1.7% (Coronavirus Disease 2020). Furthermore, they have very low morbidity or mortality from COVID-19 (Coronavirus Disease 2020). Schools were closed before description of pediatric multisystem inflammatory syndrome (Viner and Whittaker 2020). This condition has been widely reported in the media, but the facts that it is extremely rare, that it is treatable, and that almost all children will recover have been relatively overlooked. Even with what we now know about this syndrome, it is important to recognize that children remain remarkably unimpacted by COVID-19 (Viner and Whittaker 2020; Ludvigsson 2020). As of July 7th, Canada has suffered 8708 adult COVID-19 deaths but no deaths in children. Children predominantly have asymptomatic disease, but a population-wide study from Iceland showed that children were also less likely to be infected than adults (Gudbjartsson et al. 2020). One hypothesis for the low infection rate in children is the low abundance of cellular receptors for the virus (angiotensin‐converting enzyme-2) in their upper airways (Bunyavanich et al. 2020); however, other social, biological, or contextual factors may also be at play. Multiple reports have demonstrated that COVID-19 is not well transmitted in schools and that teachers were unlikely to acquire the virus from children (Pfeffer 2020; National Institute for Public Health and the Environment 2020; Danis et al. 2020). Further studies showed that children are generally not part of the transmission chain and were uncommonly the index case in family clusters (Ludvigsson 2020; Fretheim 2020; Zhu et al. n.d.). Quebec, the hardest-hit province in Canada, opened elementary schools province-wide except in Montreal, and yet there was no evidence of an increase in transmission from children (Tahariali et al. 2020).

Children being kept home have a higher risk of anxiety and depression (Xie et al. 2020), greater screen time (Media Habits of Children and Teens in Quarantine 2020), and a greater risk of physical abuse (Sidpra et al. 2020). For vulnerable families, the loss of important services from schools, including counseling, psychological services, special education, and nutritional support, is a major concern. The economic effects of parents being kept home to provide child care are particularly severe for disadvantaged families. For many of these families, childhood nutrition suffers due to loss of school meals (Dunn et al. 2020). Parents without the economic means to stay at home and educate their children because they are essential workers may not be able to keep their children at grade level. Children in Quebec from non-francophone families and children from non-anglophone families in English-speaking regions may have deterioration in their ability to speak and write comfortably in the local language, setting them further behind once classes resume. Children from marginalized groups such as immigrants, racialized minorities, and children of lower socio-economic status may find the cost of computers and internet access to be barriers that increase inequity. Preschool and children in early primary grades are most vulnerable as they often do not respond to online learning and are at a critical time of social, cognitive, and intellectual development. The long-term importance of preschool and early childhood education has been well documented with studies demonstrating effects into adulthood. A large body of research has demonstrated long-term impacts of lack of access to preschool and early childhood education, including poorer performance on intelligence, cognitive skills, and standardized tests; higher incidences of teen pregnancy and illicit drug use; lower graduation rates; lower employment rates and lower annual median earnings; higher arrest rates; and higher incidences of hypertension, diabetes, and depression (Campbell et al. 2012; Reynolds et al. 2018; Schweinhart et al. 2005; Meloy et al. 2019). Children with special needs are most severely affected as they are cut off from institutional supports. Studies from Ontario and Belgium demonstrated that even short-term school stoppages from teacher labour disruptions can lead to long-term reductions in academic achievement and a lower likelihood of completion of higher education (Johnson 2009; Belot and Webbink 2010). Although the long-term effects of our current COVID-19-related schooling policy are largely unknown, it is safe to say that there are likely to be several unintended consequences which we will recognize only after the data are compiled.

It has been argued that the policy of closing schools from March to September was necessary in order to protect the vulnerable, in particular the elderly and those with pre-existing medical conditions. This novel approach to reducing spread of infectious disease has never been adopted (in polio or scarlet fever outbreaks, school closures were enacted to protect children specifically, not some other population). Wide sweeping social policies that are likely to have a broad range of unintended consequences should be taken with great caution.

An intervention on one vulnerable group to help another should be done only in the setting of strong evidence of benefit to the other group. Initially the policy was enacted as an emergency intervention while data would accrue. However, after several months of experience with the pandemic, the benefit of school closures for other groups remains speculative. A rapid systematic review found very limited evidence of school closures having any impact on coronavirus outbreaks (Viner et al. n.d.). According to modeling studies, school closures have the greatest effect if the virus has low transmissibility (reproductive number [*R*_0_] < 2) and if attack rates are higher in children than in adults (Viner et al. n.d.), neither of which are likely true for SARS-COV-2 (Gudbjartsson et al. 2020; Tuite et al. 2020). The modeled impact of school closures was far less than other social distancing interventions (Viner et al. n.d.). A recent German study was reported to suggest that school closures helped to end the local epidemic; however, school closures occurred at the same time as most businesses were closed and therefore, the impact of social distancing among adults versus children could not be separated (Dehning et al. 2020). Taiwan successfully controlled the epidemic without widespread school closures (Everington 2020). Many alternatives to complete closures such as partial school opening have also been suggested and have recently started in Manitoba (MacIntosh 2020). Although older children (high school and above) may have higher rates of infection, preschool and elementary school children have an especially low infection risk and do particularly poorly with distant learning. Reopening schools for these younger children is an urgent priority.

In some provinces, political leaders have suggested that schools should remain closed “as we are approaching the end of the term” (CTV News 2020). This arrangement could be modified in these extreme circumstances. Perhaps the summer months would be an ideal time for children to get at least some education, especially since schools may need to close again in the fall. In fact, almost all models anticipate a second wave of infection as the autumn weather arrives, necessitating further school closures. Even a vaccine may not end the lockdown for children, as all vaccine studies are being focused at least initially on adults and so safety and efficacy studies in children will only come later. There is the possibility that if the pediatric multisystem inflammatory syndrome is due to an immunological response to viral antigens, the vaccine may be particularly harmful to children, leading to even longer delays for studying the situation before their education can resume.

The UN Children’s Fund (UNICEF) has said that the health crisis is “quickly becoming a child rights crisis” as children’s access to education, health care, and even food security is being compromised to fight a disease from which they are relatively spared (UN News 2020).

Public health officials need to continually re-evaluate the need to open or close schools as new data emerge. These new data will include the risks of new or increased frequency of complications of the virus as well as the risks of psychological, social, and educational complications from child isolation and school closure. Unfortunately, these later complications of school closures will likely take much longer to become fully apparent, and so will be appreciated only in retrospect and therefore may not get the consideration required.

In light of the limited evidence that school closures risk worsening the epidemic, and very concerning data from previous studies of long-term harm to children from school exclusion, we suggest that at least partial school opening be included as part of “phase 1” of a community opening, especially for the youngest children for whom online education is often not feasible and therefore, the long-term harms of school exclusion may be greatest. Comprehensive approaches to optimize safety, such as symptom screening prior to entry, enhanced cleaning of high-touch areas, daily disinfecting of classrooms, and prompt testing of symptomatic children and staff, have been proposed (Sharfstein and Morphew 2020).

Public health agencies and the media need to educate parents and teachers regarding the low risk associated with in-person schooling for young children in order to enable school attendance to resume. We are not advocating mandatory schooling for all, as many parents may understandably still choose to keep their children home until more data about the safety or risk of attending school become available. Teachers who are at greater vulnerability due to advanced age or comorbidities may focus on participating in remote learning for the children who remain at home. We do however emphasize that the baseline state is that children attend school for all of its well-described benefits. The state should deny children this right to education and exercise the power to over-rule parental discretion only in the setting of clear evidence that the risks of attending exceed the known risks of school exclusion, not simply “as a precautionary approach” (CTV News 2020). We need an open discussion of the ethics of mandatory closures and whether parental discretion should be overridden by the state in the present data-poor environment.
